# Folate Supplementation in Women with Pre-Existing Diabetes

**DOI:** 10.3390/nu15081879

**Published:** 2023-04-13

**Authors:** Nayomi Perera, Victoria L. Rudland, David Simmons, Sarah A. L. Price

**Affiliations:** 1 Department of Obstetric Medicine, Royal Women’s Hospital, Flemington Rd, North Melbourne, VIC 3051, Australia; nayomi.perera@mh.org.au; 2 Department of Diabetes and Endocrinology, Royal Melbourne Hospital, Grattan St, Parkville, VIC 3010, Australia; 3 Faculty of Medicine and Health, The University of Sydney, Sydney, NSW 2006, Australia; victoria.rudland@sydney.edu.au; 4 Macarthur Clinical School, Western Sydney University, Campbelltown, NSW 2560, Australia; 5 Department of Medicine, Royal Melbourne Hospital, University of Melbourne, Grattan St, Parkville, VIC 3010, Australia

**Keywords:** diabetes, pre-existing diabetes, folate, folic acid, pregnancy, preconception

## Abstract

Folate supplementation in the periconceptual period is the standard of care for the prevention of neural tube defects. To support dietary folate intake, some countries have introduced mandatory folic acid fortification of food products. Robust evidence supports the additional use of a low-dose folic acid supplement (0.4 mg/day) in all women from 2–3 months preconception until the end of the 12th week of gestation. For women with pre-existing diabetes, high-dose folic acid supplementation (5 mg/day) is recommended in some, but not all international guidelines. The recommendation is made based on consensus opinion and reflects the increased risk of neural tube defects in pregnant women with pre-existing diabetes. However, there is limited evidence to clarify the high-risk groups that benefit from high-dose folic acid versus those that do not. There are also some data to suggest that high-dose folic acid may be harmful to mothers and offspring, although this issue remains controversial. This narrative review explores the evidence that supports the recommendation for women with pre-existing diabetes to take high-dose folic acid in the periconceptual period. It explores the potential benefits of high-dose supplemental folate beyond the prevention of neural tube defects, and also the potential adverse impacts of high-dose folate use. These topics are considered with a specific focus on the issues that are pertinent to women with pre-existing diabetes. Based on the available evidence, a pragmatic approach to the use of folic acid supplements in women with pre-existing diabetes during the periconception period is suggested. The need for comprehensive preconception care that optimises glycaemic control and addresses other modifiable risk factors before pregnancy is emphasized.

## 1. Introduction

Folate is a B-group vitamin present naturally in some foods including leafy green vegetables and legumes. It is required for the body to make DNA and RNA, and to metabolise amino acids necessary for cell division [1]. Humans cannot make folate and hence it is required in the diet. Folic acid is the synthetic oxidised mono-glutamyl form of folate that is widely used in vitamin supplements and food fortification [2].

It is well established that women with folate deficiency in early pregnancy are more likely to have offspring with neural tube defects (NTDs) [3]. These defects result from the failure of the neural tube to fuse between days 21 and 27 of embryonic life. In the brain, this manifests as anencephaly or encephalocele which are generally fatal. In the spinal cord, it manifests as spina bifida which is non-fatal but results in varying levels of disability [2,4].

Around 1–2% of all women who pursue pregnancy have pre-existing diabetes which includes Type 1 diabetes, Type 2 Diabetes, and rare forms of diabetes that precede pregnancy such as monogenic diabetes and pancreatic insufficiency [5]. In women with pre-existing diabetes, the risk of an NTD is 2.2% compared with 0.57% in the general population (OR 4.2, 95% CI 2.0–7.8) [6,7]. The excess risk of NTDs in this population is largely attributable to maternal hyperglycaemia [8].

As noted in the National Birth Defects Prevention study, both maternal hyperglycaemia and folate deficiency contribute to the risk of other birth defects such as sacral agenesis, anencephaly, and cardiac defects including truncus arteriosus, atrioventricular septal defect and single ventricle complex [3,8,9,10]. It is unclear whether the co-occurrence of other metabolic diseases, such as obesity, further compounds the risk of these birth defects. The multisite case-control study of the National Birth Defects Prevention Study does not suggest a synergistic relationship between obesity and diabetes [11]. In contrast, Ray et al. found that women with multiple features of the metabolic syndrome are at higher risk of having offspring with an NTD (OR 6.1, 95% CI 1.1–32.9) than those with one feature of the metabolic syndrome (OR 1.9, 95% CI 1.1–3.4) [12].

In the context of pre-existing diabetes, birth defects are attributable to multiple factors, but suboptimal glycaemic control and folate deficiency are recognised as critical modifiable risk factors [3]. Despite the numerous international publications recommending preconceptual high dose folate in women with pre-existing diabetes, there have been no randomized controlled trials that specifically examine the optimal dose of folate for women with pre-existing diabetes [3]. Rather, existing guidelines have been developed by extrapolating data from other cohorts and are based on consensus opinion.

In this manuscript, we explore the evidence supporting the use of high-dose folate in women with pre-existing diabetes. We will review areas of controversy, highlight areas where there is a paucity of data and aim to provide clinical guidance based on existing evidence.

## 2. Methodology

We searched the MEDLINE (Ovid) online database, Cochrane Database, and the ClinicalTrials.gov registry to identify relevant studies, using the search terms “diabetes,” “pre-existing diabetes,” “folate,” “folic acid,” “pregnancy” and “preconception.” We considered for inclusion human and animal studies published in English before 1 February 2023 based on relevance, originality, and impact (e.g., number of citations). We also screened the reference lists of relevant articles. Publications reporting outcomes of clinical trials including humans were preferentially included, along with any article otherwise known to the authors relevant to the topic and not identified through the search or reference list screen.

## 3. Current Guidelines for Folate Supplementation among Women Planning Pregnancy

### 3.1. Determining the Recommended Dose of Folate Supplementation

Folic acid is generally considered safe, with an internationally-recognized tolerable upper intake level of 1 mg/day and the lowest observed adverse effect level of 5 mg/day [4,13]. The Institute of Medicine was careful to note that the upper limit for folate was developed based on the lowest level of observed effect (LOAEL) rather than the no observed adverse effect level (NOAEL) due to a paucity of evidence to establish the latter [4]. The LOAEL was set at 5 mg/day based on case studies and small observational studies demonstrating that folate doses >5 mg/day had been shown to exacerbate or precipitate neuropathy in B12 deficient individuals [4]. Although there are no long-term follow-up studies of offspring exposed to higher doses of folate, there is no evidence of direct toxicity even with folate doses of 15–100 mg/day [14,15,16]. The upper limit of folate intake from supplements or fortified food of 1 mg/day for adults was determined by dividing the LOAEL of 5 mg/day by an uncertainty factor (which is a numerical factor used by risk assessors to derive a dose considered safe; in this case, the factor was 5) [4].

Multiple studies have measured red blood cell folate in an effort to demonstrate the efficacy of folate supplementation in reducing the risk of NTDs [17]. In the original studies, models of plasma folate concentration were not presented because plasma folate was thought to be a very short-term measure of blood folate concentration, and the red blood cell folate concentration at 15 weeks gestation was thought to be the most similar to the level to which the fetus was exposed [18]. However, subsequent studies have demonstrated that red blood cell folate concentrations above 1000 nmol/L before pregnancy are associated with an optimal reduction in risk of folate-sensitive NTDs, with limited additional risk reduction seen with red blood cell concentrations above 1300 nmol/L [17]. Although both plasma folate (preferably measured fasting) and red blood cell folate concentrations are now used in research, the most accurate method of testing [19] and the utility of testing in clinical care is uncertain.

### 3.2. Folic Acid Fortification of Stable Foods

Mandatory fortification of stable foods with folic acid started in the United States in 1998 because, despite health promotion strategies, adherence to guidelines about the preconception use of folate supplements was sub-optimal. More than 80 countries have now adopted this program. In Australia, mandatory folic acid fortification of wheat flour in 2009 led to a 14.4% reduction in NTD rate (10.2 to 8.7 per 10,000 conceptions that resulted in a birth), with a 74.2% reduction in Indigenous women (19.6 to 5.1 per 10,000 conceptions that resulted in a birth) [20]. The UK and NZ mandated folic acid fortification in 2021, so pregnancy guidelines of these countries that predate these mandates should be interpreted in this context.

The Centre for Disease Control established that adults would have an estimated folate intake of approximately 0.15 mg per 100 g of fortified product, resulting in most adults consuming between 0.1 mg and 0.2 mg/day of folate [21]. When combined with a periconceptual folate supplement of 0.4 mg/day, few low-risk women would exceed the recommended 1 mg/day but equally, few women would fall below 0.4 mg/day.

### 3.3. Low-Dose Folate Supplementation as Standard Pregnancy Care

Low-dose folate supplementation refers to the use of 0.4–0.8 mg folate from 2–3 months preconception until the end of the 12th week of gestation [22,23,24]. This dose is recommended for all women by multiple international guidelines including the National Institute of Health and Care Excellence (NICE) [25], the American College of Obstetrics and Gynaecology (ACOG) [26], the Royal College of Obstetricians and Gynaecologists UK (RCOG) [27], the World Health Organisation (WHO) [28], the Society of Obstetrics and Gynaecology of Canada (SOGC) [29], and the Royal Australian and New Zealand College of Obstetrics and Gynaecology (RANZCOG) [30].

When combined with folate intake through fortified foods of 0.1–0.2 mg/day, low-dose folate supplementation generally results in a total daily folate intake of 0.5–1.0 mg/day.

### 3.4. High-Dose Folate Supplementation for High-Risk Women

High-dose folate supplementation generally refers to 4–5 mg folate per day from 2–3 months preconception until the end of the 12th week of gestation [3]. There is uncertainty about which groups of women benefit from high-dose folic acid supplementation, which has led to variations in international guidelines (See Table 1) [3]. High-dose folate (5 mg/day) is recommended for women with pre-existing diabetes by NICE [31], RCOG [27], WHO [28], and RANZCOG [30]. SOGC recommends medium-dose folate (1 mg/day) for women with pre-existing diabetes [32]. ACOG does not recommend high-dose folate for women with pre-existing diabetes and only advises high-dose folate in the setting of a previous offspring with an NTD or seizure disorder [26].

Although NICE recommends high-dose folate (5 mg per day) in women with Type 1 diabetes and Type 2 diabetes, it acknowledges that there is very little evidence to support this larger dose [33,34]. They suggest that the 4-fold higher risk of NTD in this population, compared to the general population, justifies this dosing regimen but emphasise that the increased risk of NTD is likely multifactorial [34].

In the pre-pregnancy period and during pregnancy, high-dose folate supplementation may be used in combination with a multivitamin containing 0.4–0.8 mg/day folate and folic acid fortification of food, which could result in a total daily folate supplementation intake of almost 6 mg per day.

## 4. Benefits of High-Dose Folate

### 4.1. Prevention of Neural Tube Defects

Folic acid supplementation was first proposed as a means of preventing NTDs in 1964 [35]. Subsequently, several large multicentre studies demonstrated that folic acid supplementation in early pregnancy resulted in reductions of 35–75% in the incidence of NTDs [36,37]. Mulinare et al. used data from the Atlanta Birth Defects Case-Control study and found a protective effect of periconceptual multivitamins (RR 0.40, 95% CI 0.25–0.63) [37]. Smithells et al. in 1989 subsequently published the results of a multicentre study that involved women with previous offspring with an NTD taking periconceptual folic acid 0.36 mg/day. The offspring of these women were compared with women who were already pregnant and had not used vitamin supplementation. They reported an 83–91% reduction in the recurrence of NTDs in supplemented compared to unsupplemented women [38]. Czeizel et al. reported the finding of a randomized controlled trial demonstrating that 0.8 mg/day folate reduced the first occurrence of an NTD in the offspring. These results led to the mandatory fortification of folic acid in grains and flours in many countries [39].

A 2015 Cochrane Review found high-quality evidence that periconception folic acid supplementation prevents NTDs compared to no supplementation (RR 0.31, 95% CI 0.17–0.58), but subgroup analysis showed no additional protection with higher doses of folate in the general population [21]. However, the Medical Research Council Vitamin study, a large randomized controlled trial (*n* = 1195), demonstrated that high-dose folic acid (4 mg/day) resulted in a 72% relative risk reduction (95% CI 88% to 29%) for NTD in women with a previously affected pregnancy [40]. The 4 mg dose formulation was selected because this was already available as a tablet and was not based on prior dose-finding studies. Nonetheless, the investigators thought that the 4 mg dose of folate was responsible for overcoming the causal mechanism driving the reduction in NTDs in previously affected women [3,40].

There are limited data specifically focused on the use of folate to prevent NTDs in women with pre-existing diabetes. In studies that included participants with pre-existing diabetes, the data are difficult to interpret due to the infrequent reporting of folate dose and failure to adjust for variables such as glycaemic control and body weight. Parker et al. conducted a case-control study using data from the Slone Epidemiology Center Birth Defects Study (1976–2011) and found that in women with pre-existing diabetes, lower folic acid intake (<0.4 mg/day) resulted in a lower rate of spina bifida (adjusted OR 3.95, 95% CI 1.56–10.00) than higher folic acid intake (>0.4 mg/day) (adjusted OR 1.31, 95% CI 0.17–10.30) [41]. In a subsequent case-control study using the Slone Epidemiology Birth Defects Study (1988–2015), a subgroup of women with pre-existing diabetes was examined (12 cases with NTD and 63 controls). Folic acid supplementation was associated with a lower rate of NTD (OR 0.25, 95% CI 0.04–1.05). A possible benefit from periconceptual folate doses >1 mg/day was observed but study numbers were small [42].

In summary, there is robust evidence for the use of folate to prevent NTDs. While acknowledging the increased risk of an NTD in women with pre-existing diabetes, there is limited evidence to demonstrate that high-dose folate supplementation provides additional protection compared to low-dose folate supplementation.

### 4.2. Prevention of Other Congenital Defects

In addition to the prevention of NTDs, there is some evidence that folate may prevent other types of congenital defects. This issue is particularly important given that women with pre-existing diabetes have a higher risk of other congenital defects. An initial decrease in the prevalence of severe congenital heart disease was noted after the mandatory folic acid fortification of grains in Canada [43]. This finding was consistent with a matched case-control study of almost 1800 women (600 cases and 1200 controls) in China which demonstrated that low-dose folate was negatively correlated with congenital heart disease (OR 0.60, 95% CI 0.45–0.82) [44]. Interestingly, a recent meta-analysis found that low-dose folic acid supplementation was associated with a reduced rate of congenital heart disease (OR 0.82, 95% CI 0.72–0.94). However, high-dose folic acid supplementation was positively correlated with atrial septal defects (OR 1.23, 95% CI 0.64–2.34) in this meta-analysis. This finding may reflect a baseline increased risk in the women taking high-dose folate or suggest protection from more severe cardiac defects [45].

In addition to the prevention of cardiac defects, maternal folate supplementation appears to be significantly associated with a combined decreased risk of oesophageal atresia, urinary malformations, and omphalocele in the general population (OR 0.45, 95% CI 0.29–0.71) [46]. This area has not been specifically examined in women with pre-existing diabetes.

There is strong evidence that both low and high-dose folate supplementation reduces the risk of birth defects other than NTDs. It is uncertain if high-dose folate provides additional protection compared with low-dose supplementation.

### 4.3. Prevention of Hypertensive Disorders of Pregnancy

Pre-existing diabetes is an independent risk factor for the development of hypertensive disorders during pregnancy [33]. In a meta-analysis conducted by Liu et al., pooled pregnancy outcomes in over 300,000 women with and without folate supplementation showed that the risk of gestational hypertension was not associated with folate use (RR  1.19, 95% CI 0.92–1.54, *p* = 0.19) but the risk of preeclampsia was significantly reduced (RR 0.69, 95% CI 0.58–0.83, *p* < 0.01) [47]. Subsequently, the Folic Acid Clinical Trial (FACT) was a large multicentre double blinded randomized controlled trial that aimed to determine the efficacy of high-dose folic acid supplementation (4 mg daily from 8–16 weeks gestation) for the prevention of preeclampsia in women with at least one risk factor such as pre-existing diabetes. In this study, high-dose folic acid had no benefit for the prevention of preeclampsia (RR 1.10, 95% CI 0.90–1.34, *p* = 0.37) [48].

Therefore, there is no evidence that low or high-dose folate supplementation in pregnancy protects against hypertensive disorders in pregnancy, including preeclampsia.

## 5. Concerns Regarding High-Dose Folate Use

### 5.1. Cancer Risk

There have been conflicting clinical trials regarding the impact of high-dose folate on long-term cancer risk. Charles et al. suggested an elevated risk of breast cancer and all cancer death in women who used folate during pregnancy. The group linked National Health Services Central Registry data (until 2002) with women who participated in a folate supplementation study in 1966–1967 using low-dose folate (0.2 mg/day), high-dose folate (5 mg/day) or placebo in pregnancy [49]. However, findings were only based on a small number of breast cancer cases (*n* = 31) [49]. A subsequent analysis of National Health Services Central Registry data (until 2013) found no evidence of excess morbidity or mortality from either low-dose folate (0.2 mg) or high-dose folate (5 mg) compared with placebo. Specifically, there was no increased risk of mortality from breast cancer in women who had taken 5 mg of folate per day during pregnancy [50].

In a Norwegian population-based cohort study comprising 429,004 women, 3781 cases of cancer were registered on the Cancer Registry of Norway during 7 years of follow-up. Folic acid supplementation (0.4 mg/day) before and/or during pregnancy for one (HR 1.08, 95% CI 1.00–1.18) and two or more (HR 1.06, 95% CI 0.91–1.22) pregnancies had no overall impact on overall maternal cancer risk [51].

Most recently, a systematic review exploring the relationship between maternal folate use and childhood cancer showed a protective association between folate use and acute lymphoblastic leukemia (OR 0.75, 95% CI 0.66–0.86) [52,53,54] but not acute myeloid leukaemia (OR 0.70, 95% CI 0.46–1.06) or childhood brain tumours (OR 1.02, 95% CI 0.88–1.19) [55]. Overall, folic acid supplementation in pregnancy appears to reduce the risk of leukemia in childhood [56,57] but the dose and the impact of other multivitamins are less certain.

The association between folate use in pregnancy and cancer is tenuous but cannot be completely discounted. Importantly, there is as much data to support the cancer-protective effects of folate as there is to suggest cancer-related harm.

### 5.2. Autism and Neurodevelopmental Disorders

There is robust evidence that maternal use of low-dose folate in pregnancy has a protective effect against the development of autism in the offspring. In 2012, Schmidt et al. reported the findings of the CHARGE study which demonstrated that a mean daily folic acid intake of ≥600 μg (compared with <600 μg) during the first trimester of pregnancy was associated with reduced autism risk (adjusted OR 0.62, 95% CI 0.42–0.92, *p* = 0.02). Furthermore, the risk estimate decreased with increasing folic acid dose (*p*-trend = 0.001) [58]. These findings were replicated in the larger Norwegian Mother and Child Cohort Study (MoBa) which followed up 85,176 children from birth to around 10 years of age and found that autism developed in 0.10% (64/61,042) of children whose mothers who took folate compared with 0.21% (50/24,134) in those unexposed to folic acid [59]. In 2019, a meta-analysis including 756,365 children aged 11 months to 15 years from 10 countries demonstrated that maternal folate use reduced the risk of autism by around 58% compared with children of mothers who did not take folate [60].

In contrast, the association between high-dose folate use in pregnancy, as is recommended in women with pre-existing diabetes, and autism has been less clear. In 2014, Barua et al. reported that high-dose folate supplementation in pregnant mice can alter neurodevelopmental outcomes [61]. Notably, the dose of folate and duration of use were not equivalent to the high-dose folate used in human pregnancies. In 2018, Raghavan et al. published a prospective cohort study in 1257 mother-child pairs, to examine the relationship between supplemental folate dose and the risk of autism. They found that extremely high concentrations of maternal plasma folate (>60.3 nmol/L) were associated with an increased risk of autism [62]. However, a subsequent study by Raghavan found that only high concentrations of unmetabolized folic acid in offspring of certain ethnic groups were associated with autism, but not total folate and not in all ethnic groups [63]. This suggests that the association is uncertain and requires further research.

In summary, low-dose folate supplementation in pregnancy protects against autism in the offspring, but the impact of high-dose folate remains uncertain.

### 5.3. Insulin Resistance

For women with pre-existing diabetes, especially Type 2 Diabetes, the association between high-dose folate and insulin resistance is particularly pertinent. This association has been noted in numerous studies using animal models [64,65,66,67], but the findings in human studies are inconsistent. When high-dose folic acid (40 mg/kg) was administered to women for their entire pregnancy, increased glucose titres on the glucose tolerance test and increased birthweight were observed, compared to women taking standard-dose folic acid supplementation (2 mg/kg) [68]. In contrast, Chen et al. noted that for women without pre-existing diabetes who used folate supplementation in pregnancy, there was no association between folate use and the development of gestational diabetes after adjusting for confounding factors [69]. Asbaghi et al. conducted a systematic review and meta-analysis on the impact of folic acid on glycaemia as measured by a variety of parameters including fasting blood glucose (FBG), fasting insulin, haemoglobin A1c (HbA1c), and homeostatic model assessment for insulin resistance (HOMA-IR)) in women with and without pre-existing diabetes. In total, 34,646 women were included in 21 studies. Folic acid supplementation reduced fasting blood glucose, fasting insulin, and HOMA-IR without altering HbA1c. Subgroup analysis showed that glycaemic improvement was greater when high-dose folate supplementation (5 mg/day) was used rather than lower doses of folate supplementation (<5 mg/day). However, improvements in glycaemia were very modest and may be not clinically relevant [70,71].

In summary, there is no consistent evidence that low or high-dose folate use in pregnancy causes clinically significant differences in insulin resistance.

### 5.4. Atopic Disease

Previous studies have suggested a possible association between maternal folate use and atopic disease [72,73]. In 2012, an observational study (484 infants) reported that infants exposed to >0.5 mg/day maternal folic acid supplementation in the third trimester were more likely to develop eczema than those exposed to <0.2 mg/day (41% eczema among those exposed vs. 27.3% among unexposed, OR 1.85, 95% CI 1.14–3.02) [74]. However, Molloy et al. subsequently measured red blood cell folate in each trimester of pregnancy and found no association between maternal red blood cell folate levels and the development of atopic disease including eczema [75]. There are inconsistent findings regarding the role of periconception folate use and asthma. A 2013 systematic review and meta-analysis found no association between first-trimester maternal folic acid use (compared with no use) and asthma in childhood (summary risk estimate 1.01, 95% CI 0.78–1.30) [76]. However, a 2018 population-based study found that folic acid use in the first trimester of pregnancy amongst atopic mothers may prevent wheeze in the offspring [77].

There is no consistent evidence that folate use in pregnancy predisposes to atopic disease in the offspring, and indeed it may be protective in high-risk offspring. There is no data about the impact of folate supplementation dose.

## 6. Preconception Care

Although this review focuses on the use of supplemental folate in women with pre-existing diabetes, the importance of preconception counselling must be emphasized. Congenital anomalies in women with pre-existing diabetes are largely attributable to maternal hyperglycaemia [78,79]. Ray et al. conducted a study assessing major congenital malformations among 1192 offspring of mothers with pre-existing diabetes who had received preconception care and 1459 offspring of women who had not. The pooled rate of major anomalies was lower among preconception care recipients (2.1%) than non-recipients (6.5%) (RR 0.36, 95% CI 0.22–0.59) [78].

As stated in multiple international guidelines for the care of women with pre-existing diabetes who are planning pregnancy, preconception care should include advice on the use of periconception folate and a multivitamin containing iodine [80,81]. Glycaemic management and body weight should be optimised before pregnancy [80,81]. A medication review should be discussed to ensure the cessation of teratogenic medications [80]. Women with pre-existing diabetes should also be advised to commence low-dose aspirin at the end of the first trimester of pregnancy to reduce the risk of preeclampsia [80,82].

## 7. Ongoing Research in the Use of Folate in Pre-Existing Diabetes

There are no trials that have compared high-dose versus low-dose folic acid for the prevention of NTDs in the general population, and none that have compared the dose response in a population of women with pre-existing diabetes. To our knowledge, there are also no ongoing trials exploring the impact of folic acid dose on pregnancy outcomes in women with pre-existing diabetes. The clinical utility of measuring folate, either plasma folate or red blood cell folate concentration, is also uncertain.

## 8. Clinical Care in the Context of Uncertainty

There is robust evidence for the use of low-dose folic acid supplementation in early pregnancy to prevent NTDs in the general population. There is also robust evidence for the use of high-dose folic acid supplementation in women with a previous pregnancy affected by an NTD. The evidence for high-dose folic acid supplementation in women with pre-existing diabetes is less clear but this is the currently stated guidance from multiple international societies. This recommendation is made regardless of the duration of diabetes, degree of hyperglycaemia, diabetes complication status, and other co-morbidities.

Two authoritative bodies, the US National Toxicology Program and the UK Scientific Advisory Committee on Nutrition, convened in 2017 to review the evidence of folate and specifically to review safety issues [4]. They concluded that the totality of the evidence to date does not support safety issues with currently recommended folate supplementation advice of periconceptual low-dose folate for the general population and high-dose folate for high-risk groups [4].

On this basis, the most pragmatic approach for women with pre-existing diabetes may be to use folate supplementation, including from all multivitamins and food fortification, with a cumulative upper dose of 5 mg/day from 2–3 months preconception until the end of the 12th week of pregnancy [3]. This advice applies to women planning both spontaneous and assisted conception and should be delivered as part of a preconception counselling package that also provides advice on the intake of a folate-rich diet and optimisation of glycaemic control, weight, and blood pressure.

Given the fortification of foods with folate (containing approximately 0.15 mg/day) and the broad recommendation for women planning pregnancy to take an antenatal multivitamin containing 0.4 mg–0.8 mg/day, most women will achieve a folate intake of 0.5–1.0 mg/day. Women with pre-existing diabetes could be advised to take an additional 4 mg folate tablet or half of a 5 mg tablet per day (or 5 mg taken every second day), with dosing individualised based on the risk factors of the woman. This advice would result in a total intake of 3–5 mg/day without exceeding the recommended 5 mg/day dose.

After the end of the 12th week of pregnancy, folate supplementation could be ceased or reduced to that contained in a standard antenatal multivitamin. If pregnancy does not occur after 6–12 months of trying to conceive on high-dose folate supplementation, there is no evidence to guide the ongoing use of high-dose folate supplementation. It may be reasonable to swap to low-dose folate until conception occurs and then to recommence high-dose folate until the end of the 12th week of gestation [3,32]. Importantly, this strategy has not been tested in a randomized controlled trial and it could mean that higher dose folate supplementation does not occur until after organogenesis is complete.

## 9. Conclusions

Women with pre-existing diabetes have a higher risk of NTDs than the general population. There is strong evidence to support the use of low-dose folate for both the reduction in NTDs and potentially the reduction of other congenital defects. The evidence of harm from high-dose folate is not supported by robust data and confounders must be considered in the analysis of data. In a high-risk setting, especially if a previous pregnancy has been affected by an NTD, high-dose folate is recommended. For women with pre-existing diabetes, high-dose folate is recommended with a cumulative total daily intake of up to 5 mg/day. This advice should form part of a preconception plan that is tailored to the individual woman. Further research is required to provide robust evidence to support this advice.

## Figures and Tables

**Table 1:** International recommendations for folate supplementation in women with pre-existing diabetes.

Organisation	Year	Recommendation on Folic Acid Supplementation for Women with Pre-Existing Diabetes
World Health Organization [28]	2007	5 mg/day and increased food intake of folate
National Institute for Health and Care Excellence (NICE), United Kingdom [31]	2016	5 mg/day when planning pregnancy and in early pregnancy
Royal College of Obstetricians and Gynaecologists, United Kingdom [27]	2010	5 mg/day for ≥1 month before conception until 12 weeks of gestation if BMI ≥ 30 kg/m^2^
Royal Australian and New Zealand College of Obstetrics and Gynaecology (RANZCOG) [30]	2021	5 mg/day for one-month preconception and the first 3 months of pregnancy
Society of Obstetrics and Gynaecology of Canada [32]	2015	1 mg/day with consideration of red blood cell folate level (1.0 mg if red blood cell folate ≤ 906 nmol/L and 0.4 to 0.6 mg if red blood cell folate > 906 nmol/L)
American College of Obstetricians and Gynaecologists, United States of America [26]	2020	0.4 mg/day (standard care)

## Data Availability

No new data were created or analyzed in this study. Data sharing is not applicable to this article.
